# A Computational Model of Tonal Tension Profile of Chord Progressions in the Tonal Interval Space

**DOI:** 10.3390/e22111291

**Published:** 2020-11-13

**Authors:** María Navarro-Cáceres, Marcelo Caetano, Gilberto Bernardes, Mercedes Sánchez-Barba, Javier Merchán Sánchez-Jara

**Affiliations:** 1 Department of Computer Sciences, University of Salamanca, Pza de los Caídos, s/n, 37007 Salamanca, Spain; 2 Schulich School of Music & CIRMMT, McGill University, 555 Sherbrooke Street West, Montréal, QC H3A 1E3, Canada; marcelo.caetano@prism.cnrs.fr; 3 Aix Marseille Univ, CNRS, PRISM (Perception, Representations, Image, Sound, Music), Marseille, France; 4 Faculty of Engineering and INESC TEC, University of Porto, 4200-465 Porto, Portugal; gba@fe.up.pt; 5 Department of Statistics, University of Salamanca, Pza de la Merced, s/n, 37007 Salamanca, Spain; mersanbar@usal.es; 6 Department of Didactics of Musical, Plastic and Corporal Expression, University of Salamanca, Calle Madrigal de las Altas Torres, 3, 05003 Avila, Spain; javiermerchan@usal.es

**Keywords:** chord progression, hierarchical tension, tonal interval space, melodic attraction, dissonance

## Abstract

In tonal music, musical tension is strongly associated with musical expression, particularly with expectations and emotions. Most listeners are able to perceive musical tension subjectively, yet musical tension is difficult to be measured objectively, as it is connected with musical parameters such as rhythm, dynamics, melody, harmony, and timbre. Musical tension specifically associated with melodic and harmonic motion is called tonal tension. In this article, we are interested in perceived changes of tonal tension over time for chord progressions, dubbed *tonal tension profiles*. We propose an objective measure capable of capturing tension profile according to different tonal music parameters, namely, tonal distance, dissonance, voice leading, and hierarchical tension. We performed two experiments to validate the proposed model of tonal tension profile and compared against Lerdahl’s model and MorpheuS across 12 chord progressions. Our results show that the considered four tonal parameters contribute differently to the perception of tonal tension. In our model, their relative importance adopts the following weights, summing to unity: dissonance (0.402), hierarchical tension (0.246), tonal distance (0.202), and voice leading (0.193). The assumption that listeners perceive global changes in tonal tension as prototypical profiles is strongly suggested in our results, which outperform the state-of-the-art models.

## 1. Introduction

Musical tension is widely associated with musical expression, particularly with expectations and musical emotions [1,2]. Most listeners are able to perceive musical tension subjectively, yet musical tension is difficult to be measured objectively [3,4,5]. Increasing musical tension is commonly linked to building stress and impending climax, while decreasing tension is linked to relaxation or resolution [5]. Musical tension is one of the most important and elusive concepts in Western tonal music. The perception of musical tension is speculated to arise from the combined interaction of several musical parameters, such as dynamics, melody, harmony, rhythm, tempo, and timbre, among others [1,5,6,7,8,9,10,11,12]. Existing models acknowledge the multidimensional nature of musical tension, but there is no consensus on the individual contribution of each musical parameter to musical tension or how these parameters interact [5,13]. For example, dynamics is known to play an important role in tension with increasing loudness associated with building tension [4,6,13] and certain timbral changes associated with modulation of tension [11,14,15]. Similarly, it is generally agreed that a suspension is tense in relation to its resolution [2] and so is dissonance in relation to consonance [8,10,16,17].

The role of harmony in musical tension has long been investigated [1,3,4,5,8,10,12,13]. Lerdahl and Krumhansl [1] refer to *tonal tension* as a specific aspect of musical tension created by melodic and harmonic motion. For example, in the tonal context, it is widely agreed [1,4,8,10] that motion away from the tonic increases tension, whereas returning to the tonic provides resolution. This article focuses on the *tonal tension* of *chord progressions*, whose fundamental role within Western tonal music has been historically acknowledged [18,19,20]. In this article, *chord* designates a set of three or more musical notes played simultaneously, and *chord progression* designates temporal sequences of chords [10]. Specifically, we are interested in how the perception of tonal tension changes over time as a chord progression unfolds. We aim to create a computational model that captures the perceived changes in tonal tension associated with a particular chord progression.

Tonal tension can be explained from the perspective of music theory [18,20] or music psychology, which includes cognitive [1,4,21,22] and perceptual approaches [16,23,24,25]. In music theory, models of tonal tension traditionally result from the interaction of tonal indicators from multiple pitch levels, such as the *tonal function* a chord plays within a particular key, their *sensory dissonance*, and the *horizontal motion* between the voices in the chord sequence [20,22,26,27]. The cognitive approach emphasizes the importance of expectations resulting from previous exposure to the tonal system [1,4,21,22]. Thus, changes in tonal tension can be attributed to musical events breaking or fulfilling these expectations [28]. Finally, musical concepts such as consonance and dissonance are commonly explained by association with perceptual phenomena [25]. For example, *roughness* has long been used to explain tonal dissonance [16,23,24]. Some authors have postulated that neither music theory nor music psychology alone is enough to satisfactorily explain tonal tension because these perspectives are conceptually complementary [10,29,30]. For example, a chord can be very dissonant and have a stable tonal function. Ultimately, we aim to develop a computationally feasible model of tonal tension that leverages the complementary nature of the music psychology and the music theory perspectives by incorporating perceptual concepts into music theoretical measures [31].

Tonal tension can be associated with specific musical events (i.e., a particular chord in a progression), short-term contexts (e.g., a preceding chord in a progression) or long-term contexts (e.g., an entire chord progression). Bigand et al. [10] investigated the perceived musical tension created by the second chord in a three-chord progression and Navarro et al. [32] furthers the previous design by considering two preceding chords in a three-chord progression. Later, Bigand and Parncutt [33] investigated tonal tension in longer chord progressions to emphasize the distinction between the tonal functions of the chords and their psychoacoustical features. Lerdahl [22] proposed a model of tonal tension based on his influential tonal pitch space [22,34], in which multiple explicit conditions expressed as distances are adopted as dimensions of his model. Furthermore, Lerdahl emphasized the importance of the long-term hierarchical context representation of functional dependencies across all preceding or succeeding chords in a progression [22]. In an earlier publication [35], a tension model which notably includes a tree representation to capture long-term functional dependencies in chord progressions based on Rohrmeier’s [27] generative syntax model has been proposed [35].

To date, Lerdahl’s model of tonal tension is one of the most influential and validated theories of tonal tension [1,4,5,10,33], combining music theory with our current understanding of music perception and cognition. Nevertheless, Lerdahl’s model is notoriously difficult to implement computationally [36]. There is no automated procedure to calculate the hierarchical structure of the chord progressions and the parameters of the model rely mostly on ad hoc tabulated values driven from empirical musical theory experience. For example, the *surface tension* parameter assigns integers between 0 and 4 to musical properties of the chords (i.e., scale degree, inversion) and estimates the final value as the sum of the individual scores. Consequently, the surface tension scale of the model is not constructed to be linear. Ideally, if chord $C_{1}$ has a surface tension value that is twice the surface tension of $C_{2}$, $C_{1}$ should contribute to add twice as much surface tension as $C_{2}$, for example.

Recently, Herremans and Chew [12] presented MorpheuS, a tonal tension model based on the *spiral array* [37], incorporating algorithmic aspects more readily apt for computational implementation. Their model use measures for cloud diameter (dissonance), cloud momentum (tonal movement between chords), and tensile strain (distance to the key). Furthermore, long-term features, such as typical movements of tonal functions or common melodic sequences, are extrapolated from statistical analysis of style-specific music corpora. However, the statistical nature of the models is more appropriate to capture linear changes that happen over time, whereas long-term dependencies such as phrase structures require modeling the intrinsic harmonic hierarchy. In a preliminary investigation, Navarro et al. [35] presented a computational model of tonal tension associated with each chord in a progression. It adapts Lerdahl’s tonal tension model [1] into a fully automated framework. To this end, they compute the parameters of Lerdahl’s model in the Tonal Interval Space (TIS) [38], a tonal pitch space where musical parameters can be captured as geometrical distances. The mathematical basis of the TIS promotes a computationally feasible model from which we can automatically compute musical information across multi-level pitch configurations such as chords and scales. Furthermore, the TIS has proven to encompass properties of musical theoretical value and of perceptual relevancy to create metrics that capture chord similarity [39,40,41,42]. The musical theoretical value stems from the adoption of the discrete Fourier transform (DFT) of pitch classes to represent tonal pitch in the TIS space. Pitch class is closely related to pitch chroma, which represents the group of all pitches associated with the same musical note in different octaves. For example, the pitch class C represents all the C notes in any octave. The properties derived from the mathematical model of the Fourier transform help to reflect empirical ratings of consonance between two pitches [31,39].

This article proposes a computational model of *tonal tension profile* of a chord progression that extends the previous work by Navarro-Cáceres et al. [35]. This tension profile can be conceptualized as a continuous curve that captures changes in tonal tension over the temporal sequence of a chord progression. The tonal tension profile accounts for dependencies across immediately neighboring chords and the overall tonal hierarchies of the progression and is computed by connecting all the instantaneous tonal tension values given by the measure detailed in our previous work [35]. The original contributions of our work are four-fold. First, our work advances a computational model that aims to capture the global tension profile of a chord progression beyond state-of-the-art models. Second, we tackle the challenging problem of automating the computation of long-term dependencies across chords in a progression as syntactic tree structures and by adopting the TIS in computing remaining local music properties. Third, the adoption of the TIS promotes a view from music theory and psychology, which has been shown to complementarily contribute to the perception of tonal tension [10,29,30]. Fourth, we unpack the relative importance of each parameter inspired by Lerdahl’s [1] in better predicting the tonal tension profile of a chord progression.

To evaluate our tonal tension profile model, we conducted two new experiments with contrasting approaches, whose designs were inspired by Farbood [5]. Both experiments use twelve chord progressions with five to ten chords, six of those from compositions and six others created for this investigation. The first experiment (Experiment 1) used a slider to collect real-time responses of perceived changes of *instantaneous* tonal tension. We asked expert musicians to move the slider upwards or downwards as they listened to the chord progressions to reflect increasing or decreasing tonal tension, respectively. The second experiment (Experiment 2) is an online listening test that aims to assess the *global* perception of changes in tonal tension associated with chord progressions. We proposed five *prototypical* curves that represent *global* tonal tension profiles and then we asked the listeners to select the curve that best represents the overall perceived changes in tonal tension of twelve chord progressions. Finally, we compare the results of the proposed model with two representative state-of-the-art models of tonal tension, namely Lerdahl’s [22] and MorpheuS [12]. The comparison with Lerdahl’s [22] model aims to explicitly assess how the representation space can affect the measurement of the *tonal tension*, whereas the comparison with MorpheuS [12] highlights the impact of tonal space representations and different musical properties from short-term contexts in capturing tonal tension.

The remainder of this paper is structured as follows. Section 2 presents an overview of the proposed approach to model the tonal tension profile of chord progressions. Then, Section 3 explains the estimation of the *instantaneous tonal tension* in the TIS [38], with a particular focus on an algorithmic procedure to (automatically) construct a tree that represents the hierarchical structure of a chord progression. Next, Section 4 describes two experiments we performed to evaluate the model. Finally, Section 5 presents the conclusions and the future work.

## 2. Overview

In this section, we present an overview of the approach we propose to model the tonal tension profile of chord progressions. Figure 1 illustrates the four levels of the problem addressed in our work. From top to bottom, the *global tension profile*, the *hierarchical structure*, the *chord progression*, the *model of tonal tension profile*, and the *instantaneous tonal tension profile*. The entry point is the chord progression because the other levels depend on it. The *global tension profile* is represented by a *prototypical curve* that represents the changes in tonal tension over time experienced by the listener after the chord progression is played. In the case of Figure 1, the global tension profile captures rising tension followed by relaxation. The hierarchical structure is constructed from the chord progression. One of the contributions of this work is the description (see Section 3.6) of a set of *analytical* rules to construct a tree from the chord progression based on the *generative* procedure proposed by Rohrmeier [27]. Between the chord progression and the instantaneous tonal tension profile, Figure 1 also shows a table with the values of the individual indicators of tonal tension, namely, *tonal distances* divided into *tonal distance between chords*, *tonal distance from the key* and the *tonal distance from the tonal function*; *dissonance*, *voice leading* and *hierarchical tension*. The final tonal tension associated with each chord in the progression is obtained as a linear combination of the model components. Finally, our proposed model fits a curve to the time-series of *instantaneous tonal tension* values to estimate the *instantaneous tonal tension profile* associated with the chord progression.

Figure 1 illustrates how to calculate the *instantaneous tonal tension* of the second chord (F Major). The measure of *consonance* only depends on the chord itself (highlighted with an ellipse in green), whereas the calculation of both *tonal distance* between two chords (red rectangle) and *voice leading* (orange rectangle) use a sliding window spanning two chords, the current (F Major), and the previous one. The measure of *tonal distances* from the key and from the tonal function uses the tonal context and *hierarchical tension* uses the tree that represents the *hierarchical structure* of the entire chord progression to compare the current chord with all its parents nodes. Finally, these six tonal indicators are linearly combined into the final *instantaneous tonal tension*. Then, the sliding window shifts forward by one chord and the *instantaneous tonal tension* value is estimated for the next chord. This process is repeated until the end of the chord progression is reached, resulting in a set of discrete *instantaneous tonal tension* estimations that comprise the *instantaneous tonal tension profile*. The aim of this work is to evaluate how well the *instantaneous tension profile* resulting from the model captures both the *instantaneous tonal tension profile* and the *global tonal tension profile* experienced by the listener. For such, we need to evaluate four components of the model, namely, the *instantaneous tonal tension profile* experienced by the listener, how it is associated with the *instantaneous tonal tension profile* predicted by the model, and the *global tonal tension profile* perceived by the listener and its association with the *instantaneous tonal tension profile* predicted by the model.

### Tonal Tension Indicators

We propose to model the *instantaneous tonal tension* *M* of a chord $T_{i}$ in a progression *P* as a combination of the following four components:

1. Tonal Distances (in Figure 1, the first three rows of the table (highlighted in red));
2. Dissonance (in Figure 1, the fourth row of the table (highlighted in green));
3. Voice Leading (i.e., melodic attraction) (in Figure 1, the fifth row of the table (highlighted in orange)), and;
4. Hierarchical tension (in Figure 1, the last row of the table (highlighted in blue)).

Our proposal incorporates all four components into a fully automated computational model that does not require manual calculations due to the representation in the TIS (see Section 2). The *tonal distances* are key for the development of a model of tonal tension. In the TIS, we measure three properties: (i) perceptual similarity between two consecutive chords, (ii) perceptual similarity between the chord and the key of the chord progression, and (iii) perceptual similarity between the chord and the tonal function. This last property measures how the chord is related to the most important chords in the tonal music: the Tonic, Subdominant, and Dominant.

The *dissonance* measures the psychoacoustic dissonance of chords in a progression from the interaction among the vertical component notes of each chord (closely related to the psychoacoustic concept of *roughness*). In our work, surface dissonance results from measuring the Euclidean distance between the chord encoded in the TIS and the center of the space.

*Voice leading* captures the melodic attraction between consecutive chords per voice. Voice leading is a horizontal measure that estimates the tension of each note in a voice according to the previous note. In our work, the number of semitones and the relationship between the notes can affect the evaluation of the voices. Therefore, we measure the voice leading in the TIS as the distance of both notes in semitones and the perceptual similarity between the notes of the two consecutive chords.

Finally, *hierarchical tension* captures the tension of the multiple levels of the musical structure by adopting tree-based structures driven from functional harmonic categories (i.e., tonic, subdominant and dominant categories) in a similar fashion to the syntactic trees from early generative linguistics [43]. We analyze the hierarchical structure by measuring the similarity between the chord we aim to evaluate all the parent chords in the tree. This process is illustrated in Figure 1 for the second chord in the progression, where the similarity between this chord and all the parent chords above it is calculated. The chords involved for this particular chord evaluated are indicated with arrows in blue.

Section 3 describes the calculation of *M* in detail. An important contribution of our work that is also explained in detail in Section 3 is the fully automated *analytical* rules to automatically extract a tree to represent the hierarchical structure of chord progressions based on Rorhrmeier’s *generative* rules.

## 3. Instantaneous Tonal Tension in the Tonal Interval Space

Equation (1) computes the tonal tension *M* of a chord $T_{i}$ in a progression *P* as

(1) $$\begin{array}{c} M\left(T_{i},P\right)=\omega_{1}\;d_{1}\left(T_{i},T_{i-1}\right)+\omega_{2}\;d_{2}\left(T_{i},T_{key}\right)+\omega_{3}\;d_{3}\left(T_{i}-T_{key},T_{f}\right)+\\ +\omega_{4}\;c\left(T_{i}\right)+\omega_{5}\;m\left(T_{i},P\right)+\omega_{6}\;h\left(T_{i},P\right) \end{array}$$

where $T_{i}$ is the *i*-th-chord of the progression *P*, and $\omega_{j}$ are constants that represent the weights of each tonal indicator. *M* is expressed as the linear combination of the tonal indicators detailed in Section 2 *tonal distance (between chords)* ($d_{1}$), *tonal distance (from the tonal interval vector (TIV) that represents the key ($T_{key}$)* ($d_{2}$), *tonal distance (from the tonal function)* ($d_{3}$), *dissonance* (*c*), *voice leading* (*m*), and *hierarchical tension* (*h*). As illustrated in Figure 1, *M* estimates the *instantaneous tonal tension* of each chord $T_{i}$ in a progression *P*, resulting in a time series $M\left(m\right)=\left[M\left(T_{1},P\right),M\left(T_{2},P\right),\cdots ,M\left(T_{m},P\right)\right]$, where *m* is the last chord in the progression *P*. *M* results from an adaptation of Lerdahl’s tonal indicators [1] calculated in the Tonal Interval Space (TIS) [31]. The next section briefly summarizes the most important features of the TIS. Refer to [31] for further information. The implementation of the model can be found at https://github.com/merismeris/tonal-tension-TIS.

### 3.1. Tonal Interval Space

The twelve pitches commonly used in Western tonal music can be represented by whole numbers from 0 to 11. Thus, the C major chord can be written as the row vector $\left[0,4,7\right]$ and the C major key as $\left[0,2,4,5,7,9,11\right]$. One inconvenience of this representation is that the number of pitches represented dictates the number of dimensions of the underlying space spanned by these vectors. In the previous example, the C major chord results in three dimensions while the C major scale results in seven. Therefore, this representation does not yield a natural way to compare different pitch configurations, such as chords and scales, for example. The chroma vector *C* is an alternative representation with twelve binary positions corresponding to the pitches comprising the chromatic scale arranged in ascending frequency. The value 1 in a specific position indicates the presence of that pitch, and 0 indicates absence. For example, the chroma vector representation of the C major chord is $C=\left[1,0,0,0,1,0,0,1,0,0,0,0\right]$ and, similarly, for the C major key, $C_{m}=\left[1,0,1,0,1,1,0,1,0,1,0,1\right]$.

An advantage of the chroma vector representation is that all vectors have twelve dimensions independently of the number of pitches present. However, distance measures commonly used in vector spaces such as the Euclidean distance do not capture musical properties of the pitches encoded as chroma vectors. Instead, Fourier analysis can be used to explore the harmonic relations between pitch classes represented as binary chroma vectors [31,39,40,41,42,44,45,46]. In particular, the TIS [31] maps twelve-dimensional chroma vectors onto six-dimensional complex-valued Tonal Interval Vectors (TIVs) *T* with the DFT. The six dimensions are weighted by empirical ratings of dyads’ consonance to enhance the perceptual basis of the space. TIVs [31] are useful to explore the properties of the tonal system because distances between TIVs capture perceptual proximity among pitch configurations and the magnitude of TIVs reflects the dissonance of the pitch configuration represented. Additionally, pitch configurations that share harmonic relations are mapped close together in the TIS. For example, the chords from the C major scale will be all mapped close to the tonal center of the C major scale. Finally, the magnitude of *T*, which is the distance of each TIV from the origin, can be used as a measure of *dissonance* [31]. Note that the TIS is capable of comparing pitch configurations with different number of pitches, such as chords with a different number of notes and even scales in the same space and, at the same time, unlike Lerdahl’s tonal pitch space [22] or Chew’s spiral [47].

In the TIS, two fundamental measures are used to compare two tonal interval vectors $T_{1}$ and $T_{2}$, namely the Euclidean distance and the dot product. The Euclidean distance $\mu \left(T_{1},T_{2}\right)$ is computed as

(2) $$\mu \left(T_{1},T_{2}\right)=\sqrt[{}]{\sum_{k=1}^{K}{\left|T_{1}\left(k\right)-T_{2}\left(k\right)\right|}^{2}}\;$$

where K=6 is the dimension of each TIV and *k* is each component of the vectors *T*. The dot product between $T_{1}$ and $T_{2}$ is calculated as

(3) $$T_{1}\cdot T_{2}=∥T_{1}∥∥T_{2}∥\cos\theta =\sum_{k=1}^{K}T_{1}\left(k\right)T_{2}\left(k\right)$$

where K=6, $∥T_{1}∥$ denotes the magnitude of $T_{1}$ computed as the Euclidean distance $\mu \left(T_{1},T_{0}\right)$ between $T_{1}$ and the center of the space $T_{0}$ using Equation (2). Finally, the angle θ is calculated from Equation (3) as

(4) $$\theta \left(T_{1},T_{2}\right)=\arccos\left(\frac{T_{1}\cdot T_{2}}{∥T_{1}∥∥T_{2}∥}\right)\;$$

Bernardes et al. [31] showed that Euclidean distances and angles calculated with Equation (2) and Equation (4) reflect music theoretical and perceptual properties of the relationship between $T_{1}$ and $T_{2}$. Although both metrics can be applied to pitch configurations on the same level (e.g., the distance between two chords) or across levels (e.g., the distance between a chord and a key), there are slight differences between them. The Euclidean distance from the center of the space and across pitch configurations has been shown to capture consonance and perceptual similarity, respectively. The arc-cosine, i.e., angles, across pitch configurations has been shown to capture the “alignment” between two pitch configurations, such as the best TIV translation or transposition of a given note or chord to a key. The latter measure is vital to objectively measure the extent to which a chord fits in the diatonic set (i.e., scale) of a key. The measures are extensively tested and validated in previous articles, such as Bernardes et al. [38], Bernardes et al. [31], and Navarro et al. [48].

Next, Section 3.2, Section 3.3, Section 3.4 and Section 3.5 present the details of the calculation of the four indicators adopted in Equation (1), followed by a description of the hierarchical tree construction in Section 3.6, and the description on the linear combination of the four indicators and their respective contribution to the indicator of instantaneous tonal tension in *M* in Section 4.

### 3.2. Tonal Distances

The tonal distances of chord $T_{i}$ capture three different distances: the perceptual distance $d_{1}$ from the previous chord $T_{i-1}$, the distance $d_{2}$ from the key $T_{key}$, and distance $d_{3}$ from the chord tonal function $T_{f}$ respectively as

(5) $$\begin{array}{c} d_{1}\left(T_{i},T_{i-1}\right)=\mu \left(T_{i-1},T_{i}\right), \end{array}$$

(6) $$\begin{array}{c} d_{2}\left(T_{i},T_{key}\right)=\theta \left(T_{i},T_{key}\right), \end{array}$$

(7) $$\begin{array}{c} d_{3}\left(T_{i}-T_{key},T_{f}\right)=\theta \left(T_{i}-T_{key},T_{f}\right), \end{array}$$

where μ uses Equation (2) and θ uses Equation (4), so $\theta (T_{i},T_{key})$ measures the degree of membership of $T_{i}$ to the main key of the full chord progression and $\theta (T_{i}-T_{key},T_{f})$ measures the similarity to the tonal function.

In the TIS, chords perceptually close to the key (e.g., its set of diatonic chords) have smaller distances from the key [31]. $T_{key}$ is represented in the TIS by the set of diatonic pitch classes, or, in other words, its corresponding diatonic scale. To measure the proximity of one chord to its tonal function in the TIS, we compute the chord function by finding the smallest angle calculated with Equation (4) from the chord $T_{i}$ and three vectors $T_{f}$ representing the diatonic chords of the tonic I, subdominant IV, or dominant V. $T_{i}-T_{key}$ is the chord using $T_{key}$ as reference. $T_{f}$ is the tonal interval vector resulting from computing the DFT from the diatonic chords that best represent the harmonic functions of tonic (we used I degree), subdominant (we used IV degree), and dominant (we used V degree), also referenced by $T_{key}$.

### 3.3. Dissonance

In the TIS, the magnitude $∥T_{i}∥$ measures the tonal dissonance *c* of the chord $T_{i}$. If *c* results in small values of $∥T_{i}∥$, the chord is very dissonant. The minimum dissonance *c* value corresponds to a single pitch class and the maximum to a pitch configuration with all classes (i.e., all 12 chromatic notes). Within this range, the dissonance *c* of chords results from the contribution of all intervallic dimensions of the space. Refer to Bernardes et al. [31] for a comprehensive description of the algorithm.

### 3.4. Voice Leading

Equation (8) computes the voice leading *m* of chord $T_{i}$ from the previous chord $T_{i-1}$. We consider two properties to capture voice leading between chords, namely the stability between the notes and the number of semitones between the notes. Then, *m* is calculated as

(8) $$m\left(T_{i},P\right)=\sum_{l=1}^{V}\frac{1}{e^{0.05s\mu (T_{n_{l_{i}}},T_{n_{l_{i-1}}})}},$$

where *V* is the number of voices, *s* is the number of semitones between the *l*-note of the inspected chord $n_{l_{i}}$ and its corresponding *l*-note in the previous chord $n_{l_{i-1}}$. To compute the stability of the notes from a chord in a progression, we measure the perceptual distance μ between notes $n_{l_{i}}$ and $n_{l_{i-1}}$.

### 3.5. Hierarchical Tension

Equation (9) computes the hierarchical tension *h* following the conceptual basis of Lerdahl [22]. It accounts for the distance from neighboring chords in the hierarchical tree structure of the progression *P* as

(9) $$h\left(T_{i},P\right)=\frac{\sum_{k=j}^{N}\mu \left(T_{i},T_{k}\right)}{N}\;$$

where *N* is the length of the path between the chord node and the root, $T_{j}$ is the parent chord of $T_{i}$, and $T_{k}$ is the parent chord of $T_{k-1}$ in the hierarchical tree structure of the chord progression *P*, and μ is the Euclidean distance between two chords computed with Equation (2). The computation of the hierarchical tension depends on the hierarchical structure (the tree) associated with the chord progression with the specific parent chords of the tree, as is shown in Figure 1. The next section explains in detail how the tree and the parent nodes are calculated.

### 3.6. Computing Hierarchical Trees from Chord Progressions

Rohrmeier [27] proposed a generative grammar to automatically generate a *chord progression* from a starting node. In this work, we adopt Rohrmeier’s grammatical rules to invert the process and generate a *hierarchical tree structure* given a chord progression *P*. Rohrmeier’s rules can be automated and the resulting tree can be used to compute neighboring hierarchical relations in Equation (9), unlike the procedure proposed by Lerdahl [1]. The trees that Lerdahl’s uses to compute the tonal tension of a progression are manually constructed, as they are based on the particularities presented in each chord progression and the musical experience of the authors. Therefore, they do not propose general rules that can be computed to generate trees for every chord progression, unlike Rohrmeier’s proposal.

Rohrmeier’s grammar has been validated [27] and used in previous literature to construct hierarchies and analyze tonal music [49,50,51]. Lerdahl et al. [51] compared the trees created manually against the trees generated by Rohrmeier grammar, and the results suggested that Rohrmeier can capture the idiosyncrasies of musical properties quite well. Due to the success of these previous experiments, we considered Rohrmeier’s proposal as a successful approach and construct tree structures following his instructions.

According to Rohrmeier [27], the rules in Equation (10a) to Equation (10g) characterize the core behavior of a harmonic functional grammar in a chord progression. *Non-terminal symbols* TR, SR, DR, and XR represent the tonic, subdominant, dominant regions and any of the previous regions, respectively. Non-terminal symbols can be expanded into different harmonic regions following the rules in Equation (10a) to Equation (10d). *Terminal symbols* *t*, *d*, and *s* represent tonic, dominant, or subdominant chords in the sequence, respectively. Terminal symbols can be instantiated by applying Equation (10e) to Equation (10g) and cannot be further expanded.

(10a) $$\begin{array}{c} TR⟶TR\;DR \end{array}$$

(10b) $$\begin{array}{c} TR⟶DR\;t \end{array}$$

(10c) $$\begin{array}{c} DR⟶SR\;d \end{array}$$

(10d) $$\begin{array}{c} XR⟶XR;\;\forall \;XR\in \;\mathrm{non}\text{-}\mathrm{terminal} \end{array}$$

(10e) $$\begin{array}{c} TR⟶t \end{array}$$

(10f) $$\begin{array}{c} DR⟶d \end{array}$$

(10g) $$\begin{array}{c} SR⟶s \end{array}$$

To compute a hierarchical tree from a chord progression *P*, we start by replacing each chord by its respective harmonic function *t*, *s* or *d*, computed as the minimal distance to their respective diatonic triads in the TIS using Equation (4). Then, we recursively apply the grammar rules in Equation (10a) to Equation (10g) according to the three following criteria:

- To avoid unfeasible trees, first select the ‘s’ element that appears in the lowest number of rules.
- To avoid unfeasible trees, build the tree from the inner leaves and to connect them with the outer ones.
- To avoid trees with too many nodes, always apply a rule which implies a greater number of elements. If several rules respect this criterion, select one randomly.

To clarify this process, a simple hierarchical tree construction is illustrated in Figure 2. In the first stage, the chords are replaced by ‘t’, ‘s’ or ‘d’, according to their alignment with the harmonic functions in the TIS. Secondly, the ‘s’ element can be connected to the ‘d’ element by application of Rule (10c) (Figure 2c). Next, either the first or the last ‘t’ element can be connected. The last ‘t’ element is selected at random and Rule (10b) is inversely applied. Finally, the first ‘t’ is connected with Rule (10e) and Rule (10d) (Figure 2d).

At this stage, we have constructed a tree where the leaves are the specific tonal functions of each chord in the progression and the rest represent tonal regions (Figure 2e). However, to compute the hierarchical tension of a chord, we need to calculate the distances between the chord and all the chords in the progression that can hierarchically influence the tension of this chord. Therefore, all the chords should be ordered and placed accordingly in the tree structure, as illustrated in Figure 1.

According to Lerdahl’s proposal, in a tree structure, a chord can become a parent chord when it is the most stable chord among the children. This stability is related to the perceptual distance of the chord and the tonal function, and the perceptual distance between the children chord involved. Following this concept proposed by Lerdahl, we based the stability on two premises:

- Between two children, the most stable chord is the one whose degree is related to the tonal region that the parent shows.
- If two children belong to the same tonal region, the chord with the lowest tonal distance (described in Section 3.2) becomes the parent.

We iteratively replace each parent node of the tree with the least tense chord selected from its children. Thus, starting from the deepest levels of the tree (i.e., the root), each parent node is replaced and we move up to the the next level until the top level is reached (i.e., the level with no nodes, only leaves). The parent nodes with only one child are automatically replaced by the leaf chord. The parents whose children represent different tonal regions are automatically replaced by the child with the same tonal function or region as the parent to maintain the same tonal region. Finally, the node with children of the same tonal regions is replaced by the chord that gets a lower value according to its tonal distance.

A simple example considering the tree of Figure 2 is illustrated in Figure 3. Figure 3a represents the chord progression and the tree associated. Figure 3b replaces the terminal by the specific chords (G, Am, D7, and Em). Figure 3c replaces the SR and TR nodes with the only child they have. Figure 3d replaces the DR (the dominant region) with the dominant chord D7, as Am represents a subdominant function. Similarly, Figure 3e replaces TR with the tonic chord Em and not the dominant chord D7. To replace the last node, we might select either Em or G, as both are tonic chords. However, as Figure 3f shows, G is selected as the parent node because it is the least tense chord according to the tonal distance proposed in Section 3.2.

## 4. Evaluation

This section details two experiments designed to assess how well the proposed computational model of tonal tension captures the tonal tension profiles experienced by listeners exposed to Western tonal music. Experiment 1 focuses on *instantaneous* tonal tension, whereas Experiment 2 focuses on *global* tonal tension. In Experiment 1, we asked the participants to move a slider up or down while listening to the chord progressions to reflect increasing or decreasing perception of tonal tension as the progression unfolds. In Experiment 2, we conducted an online listening test that asks the participants to listen to the same chord progressions from Experiment 1 and choose a curve that best represents *conceptually* the global tonal tension profile associated with it.

We validate the proposed model of instantaneous tonal tension in Equation (1) using the curves drawn by the users in Experiment 1. We use statistical analysis to find the weights ω that reflect the contribution of each tonal indicator in Equation (1) in explaining the curves obtained in Experiment 1. Experiment 2 investigates the assumption that listeners also perceive global changes in tonal tension after listening to a whole chord progression and that this perceived tonal tension profile can be represented by a prototypical curve (shown in Figure 1). Section 4.1 describes the chord progressions adopted in the both experiments along with the criteria used in their selection. Next, Section 4.2 describes Experiment 1 followed by Experiment 2 in Section 4.3.

### 4.1. Selected Chord Progressions

Previous empirical studies [21,26] have indicated that tension profiles are influenced by musical features like rhythm, melodic contour, dynamics, etc. In this work, the focus on tonal tension requires emphasis on harmonic properties. Therefore, we selected a total of twelve chord progressions following the criteria proposed in Lerdahl’s [1] and Farbood’s [5] work, which are summarized next.

**Rhythm:** All chord progressions were regularly played one chord every two seconds to avoid the influence of rhythmic patterns on the perception of tonal tension.

**Timbre:** All chord progressions were synthesized using Finale’s default piano synthesizer to avoid the influence of timbral changes on the perception of tonal tension.

**Length:** We aim to minimize the influence of the number of chords in the perception of tonal tension, so we use chord progressions with varying numbers of chords. All chord progressions used in the evaluation have between 5 and 10 chords because the number of chords in the progression affects the perception of global tonal tension. In general, short progressions prevent the perception of the overall structure, whereas long progressions can affect the perception of hierarchical tonal structures [52].

**Number of notes:** The chords used in these experiments are constructed with four notes as is usual in Western tonal music.

**Tree structures:** The chord progressions selected correspond to different tree structures to enable the investigation of the effect of the tree structures in the ability of the model to capture tonal tension profiles. All the selected chord progressions are different from one another, containing either different chords or the same chords in a different order. Consequently, the hierarchical structure associated with the chord progressions will be different and so will the tree structures. The tree structures are automatically created by applying the algorithm described in Section 3.6.

**Keys:** In this work, we have designed progressions in different keys. However, according to Farbood [26], pitch heights can influence the perception of tonal tension. To avoid this possible bias, we tried to use keys with very similar pitches. Therefore, all the chord progressions used in the listening tests are in C minor or C major.

#### Prototypical Tonal Tension Profiles

Figure 4 shows the curves chosen to represent the *global* tonal tension profile associated with the selected chord progressions. The curves can be explained as follows:

- (a): The perceived tension increases globally.
- (b): The perceived tension decreases globally.
- (c): The perceived tension increases at first but, at some point, starts decreasing.
- (d): The perceived tension decreases at first but, at some point, starts increasing.
- (e): The perceived tension fluctuates without a noticeable trend.
- (f): None of the curves capture the perceived tonal tension profile.

The curves shown in Figure 4 were selected to visually represent general trends in tonal tension following the work of Farbood [5]. As such, these prototypical curves represent ***global*** *tonal tension profiles* meant to be interpreted conceptually. For example, (a) represents a global trend to increase. However, the perception of tonal tension might not increase linearly (rather exponentially or logarithmically, for example) and there may be perceived fluctuations in increasing tonal tension. Overall, option (a) represents increasing tension without resolution and (b) represents decreasing tension towards relaxation. Similarly, for (c) and (d), the point where the slope changes might not be exactly in the middle. Options (c) and (d) represent respectively increasing tension followed by resolution and decreasing tension followed by a buildup. Option (e) represents the case where there is no perceived global trend in tonal tension and option (f) was included to account for cases where the listener perceives a more intricate pattern of tonal tension not captured by the previous options.

The selected chord progressions should fit one of the options in Figure 4. Thus, we selected six (6) chord progressions from compositions in Western tonal music that correspond to different musical styles but follow the tonal standards similarly. We selected the following compositions by Bach, Mozart, Shumann, Chopin, and Brahms as representative examples of Western tonal music (the harmonic reduction of these excerpts are shown in Figure A1:

- Progression 1: “Ach wie Fluchtig, ach wie nichtig” BWV 26, by J. S. Bach.
- Progression 2: “Piano sonata n. 9 in D major” K. 311 by W. A. Mozart.
- Progression 3: “Prelude” op. 28 n. 4 by F. Chopin.
- Progression 4: “Warum” op. 12 n. 3 by R. Schumann.
- Progression 5: “Drei Intermezzi” op. 117 by J. Brahms.
- Progression 6: “Claire de lune” by C. Debussy.

Additionally, we used the theoretical rules of tonal music to construct six chord progressions (Progressions 7–12) whose tension profiles correspond to one of the curves shown in Figure 4. This correspondence was made empirically, by considering that chords that usually represent tonic functions will be associated with relaxed points in our tension profile, while dominant chords will represent high points of the tonal tension profile. We analyze different configurations of chord progressions, considering the consonance of the chords, the position of the notes for the voice leading, and the hierarchical structures that they represent. The final chord progressions that we used in our experiments are shown in Appendix A (Figure A1 and the MIDI files can be accessed in https://github.com/merismeris/tonal-tension-TIS). Our hypothesis is that these five *global tonal tension profiles* are able to represent the prototypical curves categories associated with the selected chord progressions. Option (f) None was included among the options in Experiment 1 to test this hypothesis. We further hypothesize that longer musical passages may have more intricate associated tonal tension profiles that may be expressed as combinations of these basic categories. For example, (a) followed by (b) followed by (a), as in the work of Farbood [5]. It is out of the scope of this work to investigate longer chord progressions than the maximum 10 chord limit.

### 4.2. Experiment 1: Instantaneous Tonal Tension

The goal of this experiment is to analyze how the proposed model captures *instantaneous* tonal tension defined in Equation (1) as the linear combination of the tonal indicators. We collected judgments of changes in instantaneous tonal tension in real-time (Section 4.2.2), which we then process (Section 4.2.4) and compare with the instantaneous tension profile values proposed by the model and determine the influence of each tonal indicator in Section 3. We compute the weights $\omega_{j}$ in Equation (1) that best explain the judgments drawn by the participants and we also compare the results with other proposals.

#### 4.2.1. Interface of Experiment 1

Experiment 1 uses the interface shown in Figure 5 to collect the instantaneous tension profiles. On the left, there is a control panel to play the chord progressions, to advance to the next chord progression, and to reset the experiment by erasing all the drawn curves. The participants use the vertical slider in the middle to draw the tonal tension curve as they listen to the progression. The vertical axis of the Curve panel is internally mapped from 0 (minimum tension) to 100 (maximum tension). These values are hidden from the participants, who only see *more tension* or *less tension*. The participants were instructed to click “Play” to start listening to the chord progression. During playback, the participants were instructed to move the slider up when the tension increases or down if tension decreases. At the end of the chord sequence, the final instantaneous tonal tension profile will be plotted with a curve on the right of the interface. The listener can repeat the sound example by clicking “Play” after the chord progression finished playing. Each participant draws one curve for each one of the 12 chord progression. The chord progressions were presented in a randomized order and the participants were free to listen to each chord progression as many times as they wished before recording their responses with the slider.

Experiment 1 requires installation of the interface and additional explanations of how to proceed, which increased the complexity of the experiment to be done online. Consequently, we decided to perform the experiment in person or via video conference using a video tutorial to introduce the interface. In addition, we involved participants with at least six years of musical training to ensure a greater understanding and ability to perceptually identify the components of tonal tension within the Western tonal system. This experiment was done by a total of 15 participants and can be accessed online at https://github.com/merismeris/tonal-tension-TIS.

#### 4.2.2. Raw Data Collection

Figure 6 shows one of the instantaneous tonal tension profiles drawn by one of the participants to illustrate the raw data collection procedure. In Figure 6, the round markers represent the samples of the position of the slider in time, with the vertical axis corresponding to the slider scale and the horizontal axis corresponding to time in seconds. The interface collects samples of the position of the slider at a regular rate of five samples per second (i.e., 5 Hz). The duration of each synthesized chord is 2 s, so there are ten samples of tonal tension between pairs of chords. During the experiment, some participants used the entire vertical range when moving the slider while others restricted the movement to a small vertical region. Given the nature of the interface, we supposed that the vertical range used by the participants does not reflect the level of tension due to the difficulty of the task and the high cognitive load required to perform it. Therefore, we normalized all the values of the curves obtained between 0 and 1.

#### 4.2.3. Analysis of Participant Agreement

At this point, each chord progression has 15 instantaneous tonal tension profiles associated whose values are normalized between 0 and 1, each curve representing how one listener perceived changes in instantaneous tonal tension. It is important to verify if all participants drew similar curves for each progression in terms of temporal variation of tonal tension. Ideally, all 15 curves capture a similar temporal pattern of tonal tension over time that can be encapsulated into one curve that will represent the tonal tension profile of a specific chord progression.

In order to demonstrate the agreement between participants, we can apply statistical tests like kappa or ANOVA. As the data collected from the participants are numbers, we selected ANOVA, as it is specifically indicated when we have numerical data. To apply ANOVA, we have to check that the samples are independent, and that all the samples have the same variance (there exists homoscedasticity). On the one hand, as the chord progressions are totally different, we can state that the samples are independent, therefore we comply with the first condition. On the other hand, to test for homoscedasticity, we applied Levene’s test, which verifies that the samples are homogeneous, and that we have concordance in the inter-subject variance. The null hypothesis for this test is that there exists equality of variances. The *p*-value obtained in Levene’s test was 0.883. With this value, the null hypothesis cannot be rejected and therefore we can state that the samples have similar variances.

Since the two conditions are accomplished, we applied ANOVA analysis to compare the mean of the 15 curves drawn by the users. The null hypothesis of the ANOVA test is that there is no significant difference in means. The *p*-value resulting from ANOVA was 0.469, which implies that the means are not statistically different because the null hypothesis cannot be rejected. Therefore, we concluded that the instantaneous tonal tension profiles drawn are similar and can be represented by a single curve obtained from the mean of the 15 curves.

#### 4.2.4. Processing the Raw Data

The instantaneous tonal tension profile that our model proposes gives one single value per chord, whereas the raw curves that the participants drew (illustrated in Figure 7) assigned a total of ten points to each chord. However, to apply correlation and compare curves, they must contain the same number of points. Consequently, we first need to resample the curves drawn by the participants of the experiment. With all the curves resampled, we combined all the curves into one that represents them, and compared it with the curve that our model proposed. Figure 7 illustrates the process. The small round markers of the raw curves represent the samples of the position of the slider in time, with the vertical axis corresponding to the slider scale from 0 to 1. Each curve I1, I2, *…*, I15 corresponds to what participants drew with the slider. The resampled curves are the result of merging the points of the raw curves to get a curve with one point per chord. Finally, the mean curve is obtained when we calculated the mean of the values that are located in the same *i*th position.

Figure 6 illustrates the resampling process. Figure 6 shows four chords 2 s apart with their time positions marked by a vertical downward arrow (time marker). The region between time markers is the *instantaneous tension interval* divided into two regions, a **delay** followed by a **stable** region. The local tension interval is associated with the chord at the time marker placed at the beginning of the interval. For example, the second interval in Figure 6 (with the annotations) is associated with the second chord in the progression and so are the samples contained in that interval. Figure 6 also illustrates the delay due to the reaction time, which is the time it takes the participants to move the slider after a chord plays. According to previous literature [26,53], every new chord is followed by a **delay** region (during which participants typically move the slider) and a **stable** region (during which the slider remains relatively stable). We are interested in samples in the stable region to represent the *instantaneous tonal tension* associated with each chord. Therefore, the resampling operation ignores the samples in the delay region and takes the mean of the samples in the stable region. The segmentation procedure uses the difference between adjacent samples to approximate the time derivative of the samples of the slider position. The stable region is defined to be the contiguous region where the derivative is in the interval ±0.005. Each point of the resampled curve represents might take a value from 0 to 1. Finally, each *i*th point of the mean curve is the result of calculating the mean of the points that are located in the *i*th position of the resampled curves.

#### 4.2.5. Comparing the Instantaneous Tonal Tension of the Participants and Our Model

In this section, we compare the mean curves of each chord progression with the instantaneous tonal tension profiles proposed by our model *M*, which measures the tonal distance to the previous chord, the tonal distance to the key, the tonal distance to the function, the dissonance, the voice leading, and the hierarchical distance. First, we aim to analyze which features influence the perception of the tonal tension. For this purpose, we calculated the Pearson correlation to know if all the musical features are related to the tonal tension perceived.

We calculated the correlation with all the musical properties, by combining them in any possible way to know which combination of features can capture the instantaneous tonal tension profile of the chord progressions best. Finally, the model with the best adjustment included four properties, namely, the tonal distance to the key, the dissonance, the voice leading, and the hierarchical tension. The Pearson correlation ρ for this combination is 0.750.

The statistical results discarded the tonal distance between consecutive chords and the distance between the chord and its tonal function. In Equation (1), *M* computes the tonal distance of a given chord *i* from the previous chord i−1. This local indicator may not capture well the notion of tonal tension, as the perceptual distance between two consecutive chords in a tonal chord progression tends to be small. Therefore, when computing the distance between two chords associated with either high or low tension, this metric will equally result in small distances. For example, the distance between two dominant chords, typically associated with high tension, results in a very small perceptual distance. Conversely, the hierarchical tension adopts the same Euclidean distance as in the tonal distance between consecutive chords and yields statistically significant results. We believe that the difference relies on computing the metric across the hierarchical tree structure of the chord progression. The long-term context of the latter tree construct compares a given chord with all remaining chords in a progression from the above hierarchies. Therefore, the resulting distance considers the distance of a given chord to the entire range of adopted chords.

The tonal distance between a chord from its tonal function captures the stability of chord within the tonic, subdominant and dominant functions. We expected the metric to contribute to disambiguate subtle differences across multiple chords which share the same function (for example, between the dominant triad and the dominant seventh). However, this relationship might be misleading in capturing tonal tension, as the finer degrees of functional stability must be observed under a larger context. For example, the distance between a stable and unstable tonic can be relatively similar to the distance between a stable and unstable dominant. While resulting in similar values, the difference between the two tonic and dominant functions is expected to be different in terms of tension.

We calculated the weights $\omega_{j}$ for each statistically significant property by applying multiple regression, considering the degrees of freedom for the calculations. The weights and the statistical results are shown in Table 1. The $R^{2}$ for the multiple regression is of 0.563. Table 1 shows that our model *M* can capture 56.3% of the curves depicted by the participants.

#### 4.2.6. Comparison with the State of the Art

We also compared the mean curve depicted by most participants against the curve that other proposals in the literature predicted. We selected two prominent proposals to compare with: Lerdahl’s model of Tonal tension [1] and Herremans’ model of tonal tension [12], dubbed MorpheuS. The comparison against Lerdahl’s influential model of tonal tension aims to validate the Tonal Interval Space as a valid representation space to capture musical properties. Additionally, to demonstrate that the hierarchical structure influences the perception of tonal tension, we also compare the measure *M* from Equation (1). MorpheuS measures different musical properties with a different topological space. This comparison allows for assessing how both the selection of musical properties to measure and the representation space can influence the subjective ratings.

Similarly to the calculation of weights for our model described in Section 4.2.5, we calculated the weights for the tonal indicators from Lerdahl’s model that best adjust to the mean curves obtained in Experiment 1. The combination of features that best explain the tonal tension in Lerdahl’s model includes the hierarchical tension and the dissonance, excluding the perceptual distance and the harmonic attraction (or voice leading), with a Pearson correlation ρ of 0.644. Table 2 presents the weights corresponding to each feature obtained when we applied multiple regression. The correlation ρ of Lerdahl’s model with these weights is 0.677 and the $R^{2}$ for the multiple regression is of 0.458. Thus, Lerdahl’s model can capture 45.8% of the curves that represent the instantaneous tonal tension perceived by the participants of Experiment 1.

The available implementation of MorpheuS computes the final value of tonal tension as a combination of features for the centroid, diameter, and the tensile strain properties [12]. In this case, we also applied multiple regression to calculate the weights with the best correlation ρ and the $R^{2}$ adjustment and compare the curve that MorpheuS predicted against the mean curves of Experiment 1. In this case, the three properties are significant to capture the tonal tension profile of the musical excerpts. The value of Pearson correlation ρ was 0.700 and the adjustment $R^{2}$ was 0.489, which means the model is able to capture 48.9% of the curves. Although these are also good results, according to the correlation ρ and the $R^{2}$ adjustment, the model that best captures the mean curves of the participants is our model, with a correlation of 0.750 and a $R^{2}$ of 0.563.

In this work, we also compare and analyze the results for each chord progression individually. Figure 8 shows the instantaneous tonal tension profiles for each chord progression. Each marker is the instantaneous tonal tension value obtained from the models *M*, Lerdahl’s and MorpheuS. Each marker is the mean of the values of instantaneous tonal tension from each participant, and the standard error (illustrated with an error bar). The rounded blue markers connected linearly with a blue semi-dotted line shows the curve that our measure *M* obtained. The instantaneous tonal tension values of Lerdahl’s proposal are represented with rounded markers and connected with an orange line-dot-dot line, whereas a dotted yellow line with rhomboid markers is the curve depicted by MorpheuS. Finally, the mean curve representing the perception of the participants is represented with a green continuous line and square markers. Visual comparison of the curves of the models against the mean curves of the participants reveal that the instantaneous tonal tension profiles that the participants perceive match the instantaneous tonal tension profile that our model *M* predicts for several cases. Therefore, to statistically validate the visual results shown in Figure 8, we calculate the Pearson correlation ρ for all the progressions tested (the rows) and the models *M* (presented in the column 2), Lerdahl’s (column 3) and MorpheuS (column 4). The highest ρ values are highlighted in boldface in Table 3, whose analysis also corroborates that *M* captures the instantaneous tonal tension profile of the chord progression quite well, as we obtained high Pearson Correlation ρ values, almost all above 0.7. As we can observe in Table 3, our measure obtained the highest adjustment ρ in most cases. According to our results, our measure better captures the instantaneous tonal tension profile for the chord progressions used in the experiments.

Interestingly, the tonal tension profile of P12 is poorly captured by all models under consideration as it shows the lowest results for adjustment. However, careful consideration of the curves resulting from the participants and the models in Figure 8 suggests that, while a fluctuating (flat) curve seems to be captured by all models, the low adjustment values may be driven from scaling factors or the magnitude changes in the models. As the progression P12 mostly includes tonic chords only, the finer degrees of stability of the chords stemming from chord inversions (given by the note in the bass), for example, are not considered in our model and MorpheuS [12].

### 4.3. Experiment 2: Global Tonal Tension

The goal of Experiment 2 is to investigate the *global* perception of tonal tension associated with the chord progressions selected in Section 4.1. We designed a listening test that asks the participants to select which curve best represents the *global* variation of tonal tension over time. Section 4.3.1 presents details about the listening test. Section 4.3.2 uses the data obtained by the participants in Experiment 1 to associate the best prototypical curve to each progression.

Both Experiment 1 and Experiment 2 are measuring tonal tension from a different point of view. Section 4.3.6 discusses the relationships between the results of the instantaneous tonal tension profile obtained in Experiment 1 and the global tension profile results obtained in Experiment 2. Finally, Section 4.3.3 compared the prototypical curves obtained in the listening test against the curves that the models M, Lerdahl’s, and Morpheus predicted.

#### 4.3.1. Listening Test Settings

Experiment 2 aimed to associate a prototypical curve to the perceived global tonal tension profile of chord progressions with an online listening test, which can be found at https://usalinvestigacion.eu.qualtrics.com/jfe/form/SV_cSChvf8II2LwlD. In Experiment 2, the participants are asked to listen to all twelve chord progressions one by one and choose one curve among those shown in Figure 4 to represent the perceived *global* tonal tension profile. The order of presentation of the chord progressions was randomized for each participant. Participants were free to listen to each chord progression as many times as they wished before selecting one of the options and they had to make a selection to proceed to the next chord progression. In total, 73 people completed the experiment. The profile of the participants was varied, we have 51 people with musical training, and 22 without any musical training. The results of the selection and the significance of the subjective ratings will be explained in Section 4.2.6.

#### 4.3.2. Raw Data Collection

Table 4 shows the selection rates (in percentage) of each prototypical tonal tension profile (columns) for each chord progression (rows). The option with the highest percentage for each particular chord progression is highlighted in bold in Table 4. Each row presents the percentage of selection of each specific chord progression. Columns 2 to 6 show the curve option according to Figure 4. As we can see, between 40% and 68% of the participants selected the same option in most cases.

In line with the results of Progression 12 in Experiment 1, the participants of the listening test have shown a smaller degree of agreement for this progression, with 19.178% of the participants unable to fit the perceived global tonal tension to any prototypical curve. The participants selection for Progression 3 equally raises the interesting question of the subjectivity and difficulty of the task at hand. Progression 3 has a surprisingly number of participants selecting contrasting ascending (46.575%) and descending (32.877%) curves. Considering the broad scope of diversity in the musical expertise of the participants in the listening test (from no training to advanced training), we believe that, in this progression, pitch height may have played an unintended impact. Despite our attempts to minimize differences in pitch height, the progression along with its highest voice unfold across two octaves. The interval of a tenth in the highest voice may have imposed a tendency for participants to select a descending curve. However, our expectation was the selection of the ascending prototypical tonal tension curve due to the tree structure that supports the chord progression. Our expectation is nonetheless verified in Experiment 1, where all the participants declared the advanced musical expertise from at least six years of musical training. Participant selection for Progression 5 also has conflicting views. The participants selected three prominent curves: descending (33.333%), convex (29.167%) curves and fluctuating (26.389%). We believe that the conflicting views across the descending and convex curves may be due to the early tension peak in the second chord of the progression. Participants may have neglected the first chord and considered that the descending line better captures the overall tension profile. However, as shown in Figure 8, an increase in the tonal tension from the first to the second chords seems evident for the participants drawn curves in Experiment 1. The selection of the fluctuating curve suggests a possible impact of pitch height, as very small differences in the outer voices of the chord progression exist. Furthermore, in Figure 8, we can observe that only the second and last chords seem to prominently denote jumps in tonal tension. Therefore, participants in the listening test may focus on the most prominent profile across time from chords 3 to 6 in which a fluctuating stable pattern seems prominent.

#### 4.3.3. Analysis of Participant Agreement

In this section, we validate the participants’ selection in the listening test by presenting their agreement in selecting a prototypical curve. If the results are positive, we will process the responses of the listening test and compare them with our model. With categorical data, we cannot apply ANOVA as in Experiment 1. In this case, we used the kappa test (κ) to test the agreement between observers. The κ coefficient shows to what extent the observers agree in their measurement, and can take values from 0, which means the matching is due exclusively to chance, to 1, which means the agreement is complete. In general, when the values are above 0.2, we can state that there is some level of agreement among the participants.

Table 5 shows the kappa coefficient (κ), the confidence intervals, and the *p*-value for each curve selection in Table 4 to quantitatively determine the statistical significance of the results. The kappa coefficients obtained are all around 0.2, which means there is a level of agreement among the participants. The *p*-values below $10^{-6}$ and the confidence intervals also support that there is a level of agreement among the participants. We conclude that the subjective evaluations given by the participants are statistically significant. Consequently, we use the curves highlighted in bold in Table 4 to represent the perceived *global* tonal tension profile associated with the chord progressions presented.

#### 4.3.4. Raw Data Processing

The model of tonal tension profile results in an instantaneous tonal tension profile associated with the chord progression. In this section, we explain how we selected which prototypical curve describes best the global tonal tension of the chord progressions. The prototypical curves are an abstraction of the instantaneous tonal tension. Therefore, we used the mean curves that the participants drew in Experiment 1 to obtain a prototypical curve that represents each chord progression. To select the prototypical curve for each chord progression, we analyzed the shape of the mean curves obtained in Experiment 1 (shown in a continuous green line in Figure 8). We applied the following tentative definitions using the first and last points as anchors to decide which curve is associated with the instantaneous tonal tension profile:

- Increasing tension profile: The points of the mean curve in Experiment 1 are higher than the first point but lower than the last point.
- Decreasing tension profile: The points of the mean curve in Experiment 1 are lower than the first point but higher than the last point.
- Concave tension profile: All the remaining points are below the first and the last points of the of the mean curve in Experiment 1.
- Convex tension profile: All the remaining points are above the first and the last points of the of the mean curve in Experiment 1.
- Fluctuating tension profile: We have points below and above the first and last points of the of the mean curve in Experiment 1.

The mean of the curves drawn for the participants can be classified into the ones shown in Table 6. Each row shows the results for one chord progression. The second column shows the prototypical curve that best describes the instantaneous tonal tension that the participants drawn in Experiment 1, according to the tentative definitions.

#### 4.3.5. Comparison of the Global Tension Profile against an Instantaneous Tension Profile

We also compared the curves associated with the ones from Experiment 1 against prototypical curves that the participants chose in the listening test. The second column of Table 6 shows the curve that most participants selected in the listening test. All the prototypical curves from Experiment 1 coincide with the most voted curves in the listening test, except in the case of Progression 5 (P5) and Progression 11 (P11). In both cases, the participants in Experiment 1 perceived the tension is going up and then down, while the participants of the listening test felt the tension is only decreasing. We have to consider that the participants of Experiment 1 drew the curves while they were listening to the chord. When they listen to the first chord, they are not able to locate the tension, as they do not know the musical context beforehand (they do not have knowledge about the scale, tonal functions, etc). This knowledge is perceived unconsciously when several chords come into play. In fact, for the case of Progression 5, if we remove the point that represents the first chord in the curve of Experiment 1, we obtain a descending line. In the case of Progression 11, the opposite may have occurred. In the listening test, the prototypical curve is assigned after listening to the whole chord progression. As the chord progression is relatively long (seven chords), the participants in the listening test might have remembered just the final part, which is a descending line, and, consequently, they selected this option. We also have to consider that, according to Table 4, the second most voted curve in Progression 5 and Progression 11 was an inverted parabola, just what the participants drew in Experiment 2, which might support our conjecture.

#### 4.3.6. Comparison of the Global Tension Profile Perceived in the Listening Test and the Models Prediction

Section 4.3.1 presented the selection rates of the participants for each chord progression. Section 4.3.4 presented the curves that our model chose. In this section, we validate if the curves selected by the model coincide with the curves that most participants selected in the listening test. We used the same definitions than in Section 4.3.4 to decide which prototypical curve is associated with the instantaneous tonal tension profile that our model predicts. Table 7 compares the prototypical curves which were selected by most part of the participants against the prototypical curves that the model proposed. The rows represent the chord progressions, while column 2 shows the prototypical curve selected by most of the participants in the listening test. Column 3 shows the prototypical curves that fit best with the definitions proposed in Section 4.3.4 according to the curve that the model *M* predicted (illustrated in Figure 8). In general, the prototypical curve associated with the instantaneous tonal tension profile of model *M* coincides with the prototypical curve voted by most participants of the listening test.

According to Figure 8, our model shows an ascending tendency in Progression 3, except for the last chord, where a downward jump occurs. A similar tendency occurs in Progression 5. It results in a descending tendency with the exception of the first point, where an upwards jump occurs. However, by strictly applying the definitions in Section 4.3.4, the curve that our model proposes is concave, even if visually we can perceive a tendency to go up (Progression 3) or down (Progression 5) except for one point in each progression. This demonstrates that the tentative definitions proposed in Section 4.3.4 do not appropriately capture the prototypical curves in all cases.

We also compared the results with our model *M*, Lerdahl’s [53] model, and MorpheuS [12]. To get the prototypical curves of both models, we again apply the same tentative definitions and select which curve best adapts to what the models predicted. Columns 4 and 5 in Table 7 show the prototypical curves of each chord progression for Lerdahl’s model and MorpheuS, respectively. Table 7 shows that the prototypical curves selected by participants in the listening test agree with the models in six out the twelve progressions. The differences in Progressions 3 in both models, Progression 5 in the case of Lerdahl’s and Progression 11 in the case of MorpheuS, can be due to the same reason than exposed with our model *M*. In short, there are some deviations in the instantaneous tonal tension profiles that are not contemplated in the tentative definitions proposed in Section 4.3.4. Future work should investigate how to objectively create prototypical curves that capture the overall tension of short chord progressions.

Table 7 also shows differences in Progressions 4 and 9. Both progressions include some non-chord tones, i.e., embellishing tones that typically exist as chromatic passages per voice. Such movements usually influence the global tonal tension perception. The lack of consideration for voice leading in Lerdahl’s model and MorpheuS led us to believe that it may have some impact on the discrepancy between the listening experiment results and the models. However, the multidimensional nature of these progressions makes it hard to pinpoint the level of contribution of each individual musical parameter that the models capture. In any case, the model that has captured best the overall tonal tension in a general case is our model *M*, which has different results only in two progressions, while Lerdahl’s and MorpheuS models differ in four occasions.

Lerdahl’s model and *M* capture the same properties but in a different topological space. In general, Lerdahl also obtains good results, but, according to the number of coincidences between the prototypical curves of the listening test and the ones predicted by Lerdahl’s, the Tonal Interval Space helps to capture musical properties better. MorpheuS also captures the tension profile with fair results. They measure linear properties, not applying any hierarchical component to capture the musical structure. By comparing the results obtained with MorpheuS and *M*, we can state that the hierarchical and long-term properties are key features to capture global tonal tension in our particular excerpts.

One of the limitations of our model is that the voice leading measure depends on an equal number of notes (i.e., voices) in the chords. There are many different styles in classical music, like Baroque, Classical, or Romantic compositions, in which the number of voices are stable in whole compositions. We were based in these styles to create our excerpts and, therefore, did not consider a variable number of voices. Future work will study how to overcome this limitation computationally in our model and how the lack of voice leading in some voices can influence the perception of tonal tension.

## 5. Conclusions and Future Work

In this work, we proposed a model that captures the *tonal tension profile* of a chord progression by encoding the chords in the Tonal Interval Space (TIS) [31]. We measure the *instantaneous tonal tension* of each chord considering factors such as dissonance, the tonal distances to measure the similarity with the previous chord, with the tonal function and with the key, voice leading, and the hierarchical tension of the chord with respect to the rest of the progression. To create the tonal tension profile, we connect the values associated with each individual chord and create a curve that represents the tonal tension profile of the chord progression. We considered the TIS as an appropriate representation for multiple reasons. Firstly, multi-level pitch configurations can be represented, such as chord with different number of notes, or keys. Therefore, we can handle chord progressions written in different keys or different modes. Additionally, topological properties of the space reflect musical properties of the tonal system. Consequently, we have constructed a flexible computational analysis framework which allows for excluding annotations on the analyzed musical data by encoding pitch configurations and capturing their musical properties systematically.

The evaluation of a chord progression depends on the musical context. This is influenced by both linear properties, related to how the progression is changing along the time and hierarchical properties, related to the structure of the whole progression. Our measure is appropriate, as it captures linear properties of the chord progressions, namely, dissonance, the tonal distances and the voice leading; and the hierarchical properties, such as the hierarchical tension of the chord with respect to the rest of the progression.

In order to evaluate how our measure predicts the tonal tension profile of a chord progression, we performed two experiments. For both, we used 12 chord progressions in different keys, with different trees (i.e., hierarchical structures), and different tonal tension profiles. Experiment 1 aimed to evaluate if our model captures the instantaneous tonal tension profile while the participants are listening to a chord progression, and which musical properties are essential to predict the tension of a progression. Experiment 2 was intended to evaluate if our measure captures the general perception of the tonal tension profile after the participants listened to a chord progression. In both cases, statistical analysis showed that the subjective ratings correlate with the objective values, validating the objective model as a proxy for the subjective measure of *instantaneous* and *global tonal tension profile* of the chord progressions proposed in the experiments. In both experiments, we also compared the results obtained with present model and with Lerdahl’s [22] and Herremans’ models [12]. Despite the positive results for all the tonal tension models, in most cases, the model *M* proposed was a better indicator of tonal tension profiles.

Finally, it is important to highlight that both global and instantaneous tonal tension are high-level properties of music, so tonal tension can be musically perceived and subjectively rated differently. Despite the limitations, one of the contributions of this model is the beginning of a generalization function that can study a long-term chord progression based on both hierarchical and linear structures in different tonalities. The adaptation of Lerdahl’s space to the TIS to explain the tonal properties of a chord besides tonal tension and voice leading can also be considered a contribution for the community. The positive results are encouraging to apply this measure to real analysis problems and computational generative systems.

Future work will study how to extend this analysis and extrapolate the model to any chord progression that has been composed following the tonal music standards. We will also analyze properties like pitch height and rhythm, which also influences the tension perception. Additionally, we will also investigate how to improve a system to generate chord progressions using an artificial immune system described in a previous work [32] with this new measure to create chord progressions that follow a given pattern, like a tension curve or a given structural tree.

## Figures and Tables

**Figure 1:**
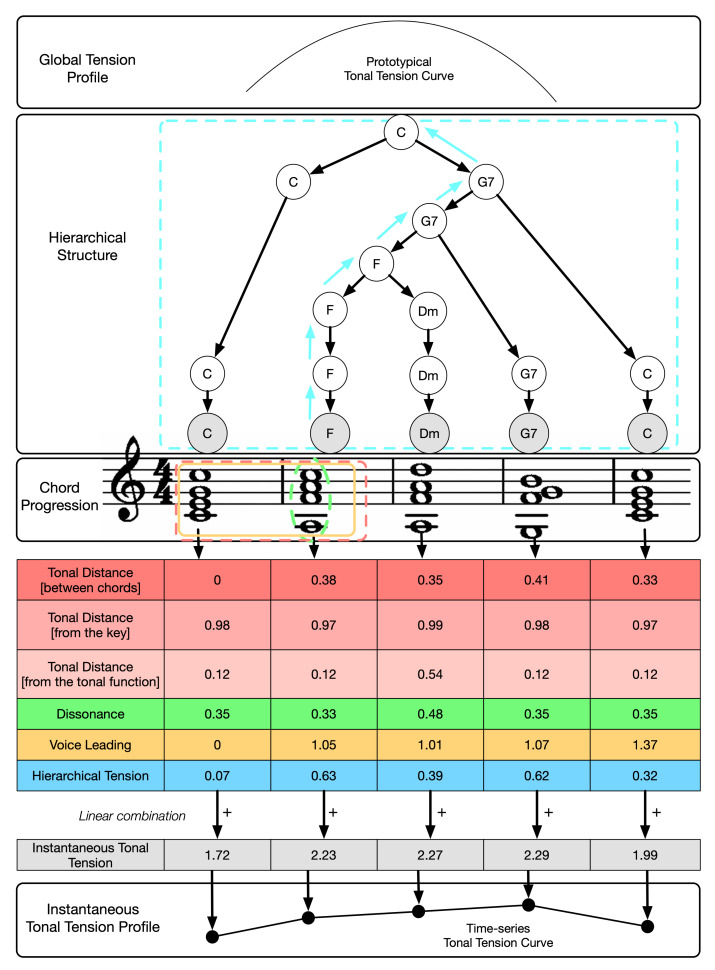
Overview of the proposed approach to model the perceived tonal tension profile of chord progressions.

**Figure 2:**
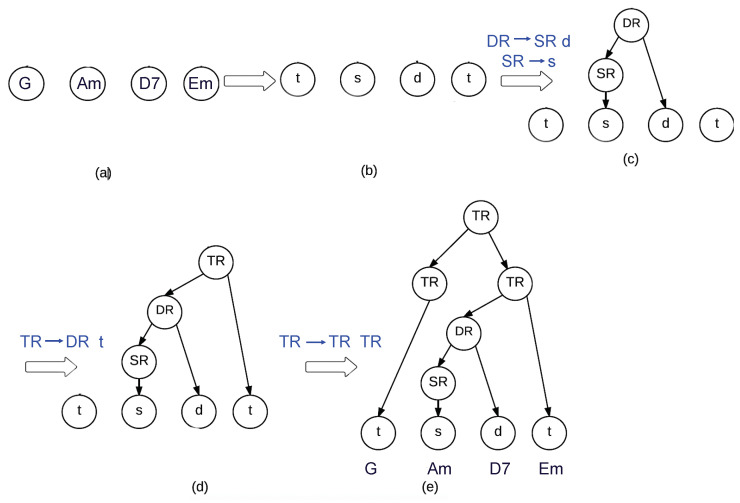
Illustration of the process to obtain a tree structure from a chord progression. Part (**a**) shows the initial stage with the chord progression; parts (**b**–**d**) show the intermediate steps while applying the grammar rules; and part (**e**) shows the final tree extracted from the initial chord progression.

**Figure 3:**
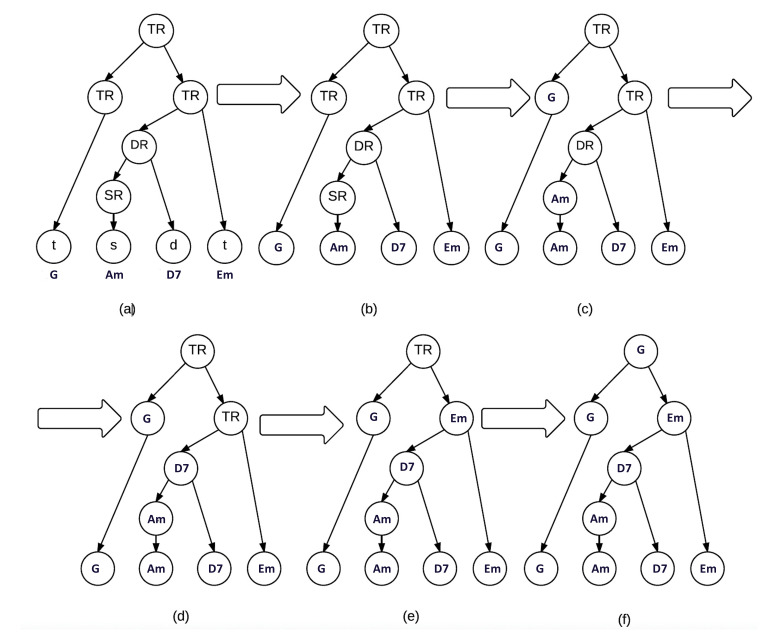
Visualization of a tree structure with specific chords to measure the tension. Part (**a**) shows the initial tree from Rohrmeier’s grammar; parts (**b**–**e**) show the intermediate steps while calculating the chords hierarchically above the tree; and part (**f**) shows the final tree with the most important chords hierarchically speaking.

**Figure 4:**
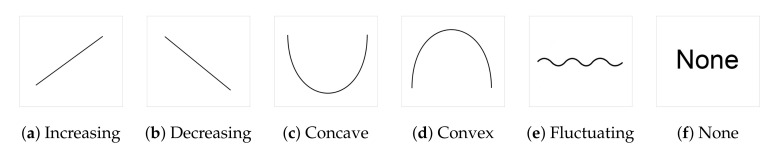
Plot of the curves presented in the listening test.

**Figure 5:**
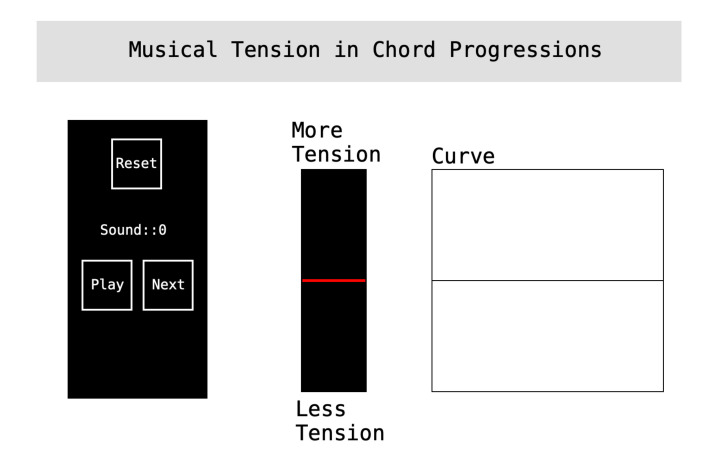
Visualization of the interface of Experiment 1 to capture the instantaneous tonal tension.

**Figure 6:**
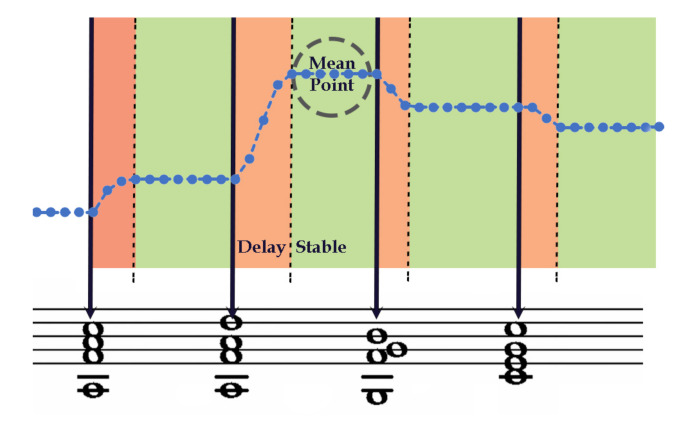
Curve that one of the participants drew during the experiment.

**Figure 7:**
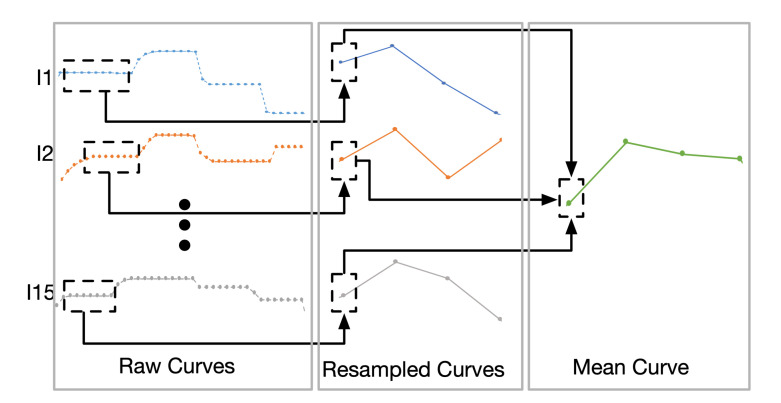
Processing of the curves depicted by the participants of the experiment and get the mean curve.

**Figure 8:**
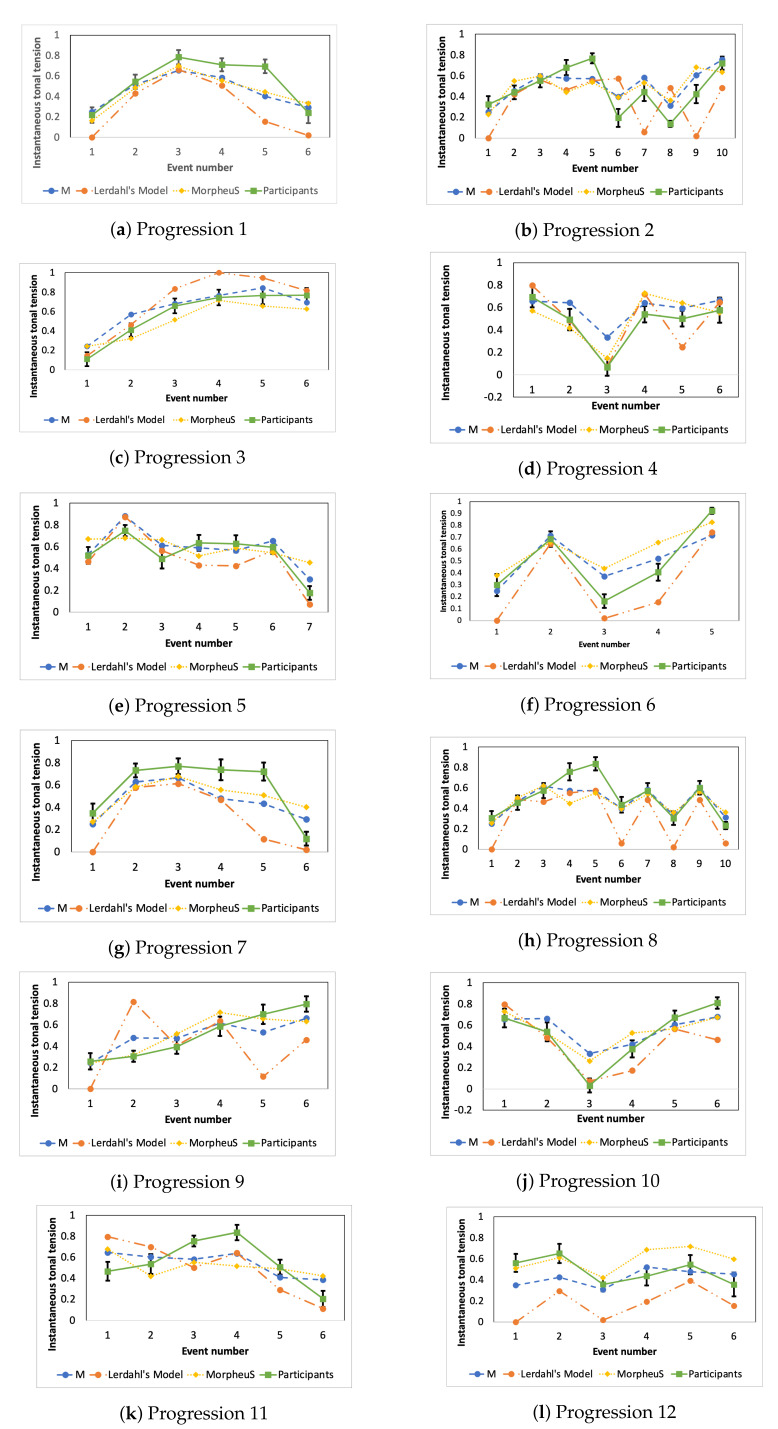
Plot of the curves predicted by the models.

**Table 1:** Weights $\omega_{j}$ of the model *M* and the statistical significance.

Feature	$\omega_{j}$	*p*-Value	95% Confidence Interval	
			Lower Level	Upper Level
Tonal distance (from the key)	0.158	0.021	−0.032	0.348
Dissonance	0.303	0.000	0.095	0.510
Voice Leading	0.271	0.042	0.101	0.441
Hierarchical Tension	0.318	0.015	0.121	0.514

**Table 2:** Weights of Lerdahl’s model and MorpheuS and the statistical significance.

Model	Feature	Weight	*p*-Value	95% Confidence Intervals	
				Upper Level	Lower Level
Lerdahl’s	Hierarchical	0.441	0.001	0.240	0.643
	Dissonance	0.323	0.001	0.122	0.525
MorpheuS	Tensile	0.488	0.000	0.318	0.658
	Centroid	−0.236	0.007	−0.405	−0.067
	Diameter	0.403	0.000	0.236	0.570

**Table 3:** Statistical analysis of the curves for each chord progression.

	Pearson Correlation ρ		
	**M**	**Lerdahl’s**	**MorpheuS**
P1	0.884	0.823	**0.899**
P2	0.778	**0.828**	0.545
P3	**0.962**	0.927	0.945
P4	**0.902**	0.853	0.827
P5	**0.848**	0.803	0.409
P6	0.881	**0.963**	0.898
P7	**0.842**	0.756	0.787
P8	0.857	**0.876**	0.698
P9	0.638	−0.117	**0.874**
P10	**0.924**	0.801	0.922
P11	**0.552**	0.529	0.271
P12	−0.062	**0.466**	0.316

**Table 4:** Frequency table that shows the percentage of votes for each chord progression (rows) and category (columns) of the participants of the listening test.

	Selection Rate (%)					
Progression	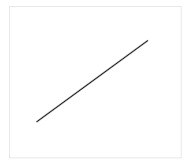	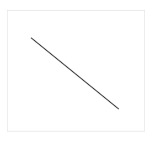	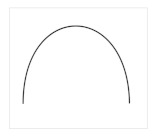	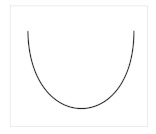	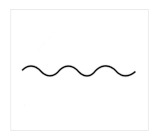	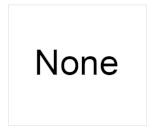
P1	6.849	8.219	**58.904**	9.589	13.699	2.740
P2	13.699	2.740	6.849	5.479	**67.123**	4.110
P3	**46.575**	32.877	6.849	1.370	9.589	2.740
P4	9.589	23.288	6.849	**41.096**	9.589	9.589
P5	1.389	**33.333**	29.167	5.556	26.389	4.167
P6	22.222	2.778	1.389	13.889	**51.389**	8.333
P7	2.740	16.438	**58.904**	15.068	4.110	2.740
P8	4.167	4.167	12.500	8.333	**68.056**	2.778
P9	**49.315**	13.699	5.479	19.178	6.849	5.479
P10	10.959	19.178	4.110	**52.055**	12.329	1.370
P11	4.167	**40.278**	27.778	4.167	16.667	6.944
P12	8.219	2.740	26.027	2.740	**41.096**	19.178

**Table 5:** Evaluation of the value, the confidence intervals and the *p*-value for κ coefficient.

Curves	κ	Confidence Intervals		*p*-Value
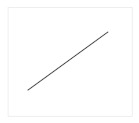	0.204	0.026	0.376	0.000
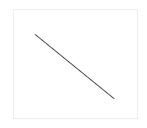	0.167	0.052	0.279	0.000
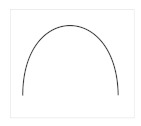	0.229	0.051	0.401	0.000
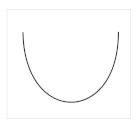	0.192	0.032	0.346	0.0000
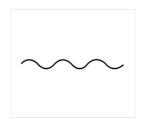	0.277	0.178	0.372	0.000
Total values	0.205	0.172	0.235	0.000

**Table 6:** Raw Data Processing.

Progression	Experiment 1	Listening Test
P1	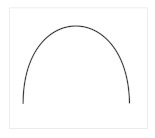	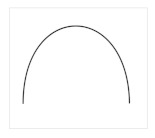
P2	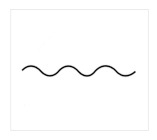	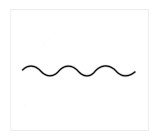
P3	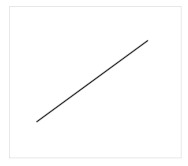	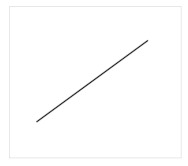
P4	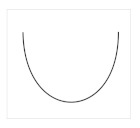	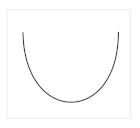
P5	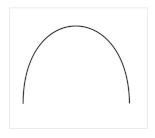	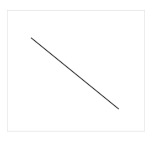
P6	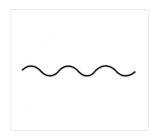	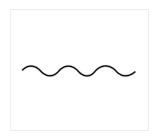
P7	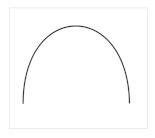	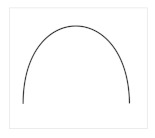
P8	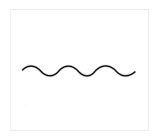	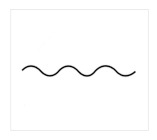
P9	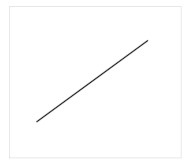	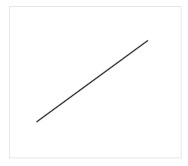
P10	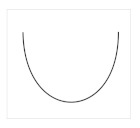	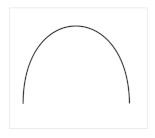
P11	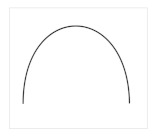	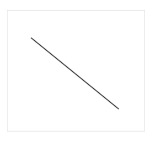
P12	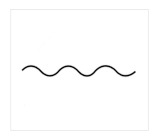	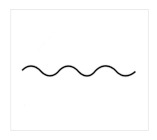

**Table 7:** Association of the prototypical curve in the listening test and our model, Lerdahl’s and MorpheuS.

Progression	Listening Test	*M*	Lerdahl’s	MorpheuS
P1	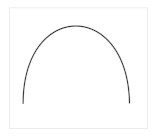	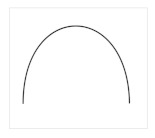	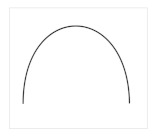	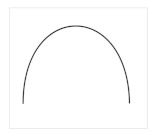
P2	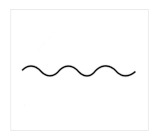	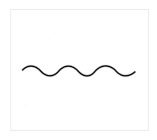	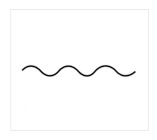	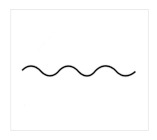
P3	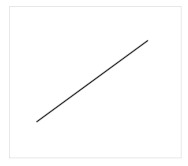	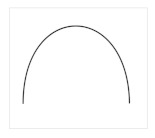	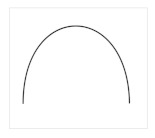	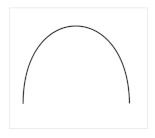
P4	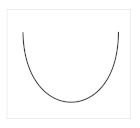	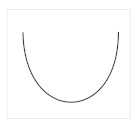	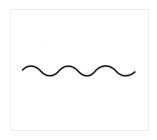	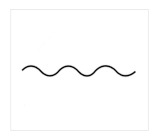
P5	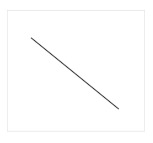	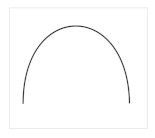	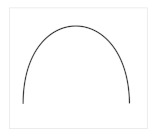	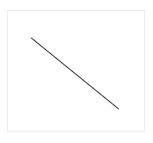
P6	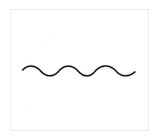	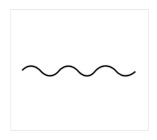	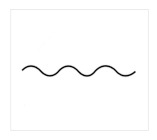	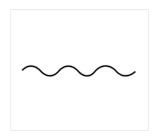
P7	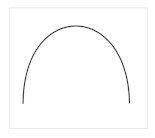	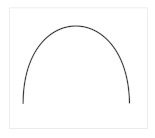	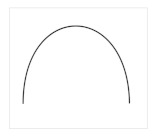	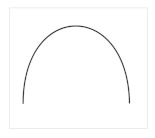
P8	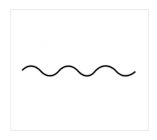	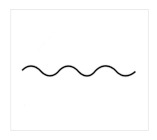	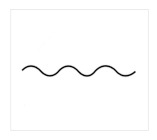	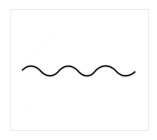
P9	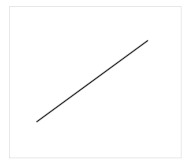	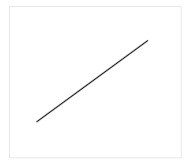	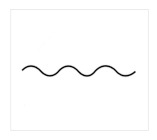	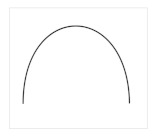
P10	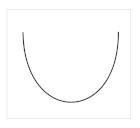	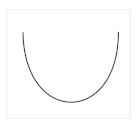	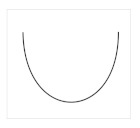	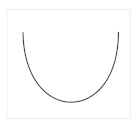
P11	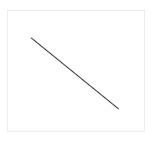	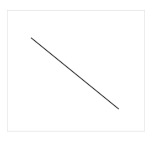	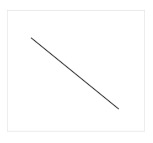	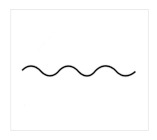
P12	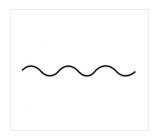	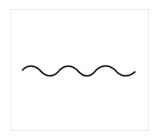	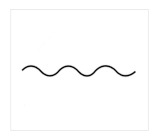	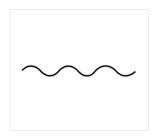

**Figure A1:**
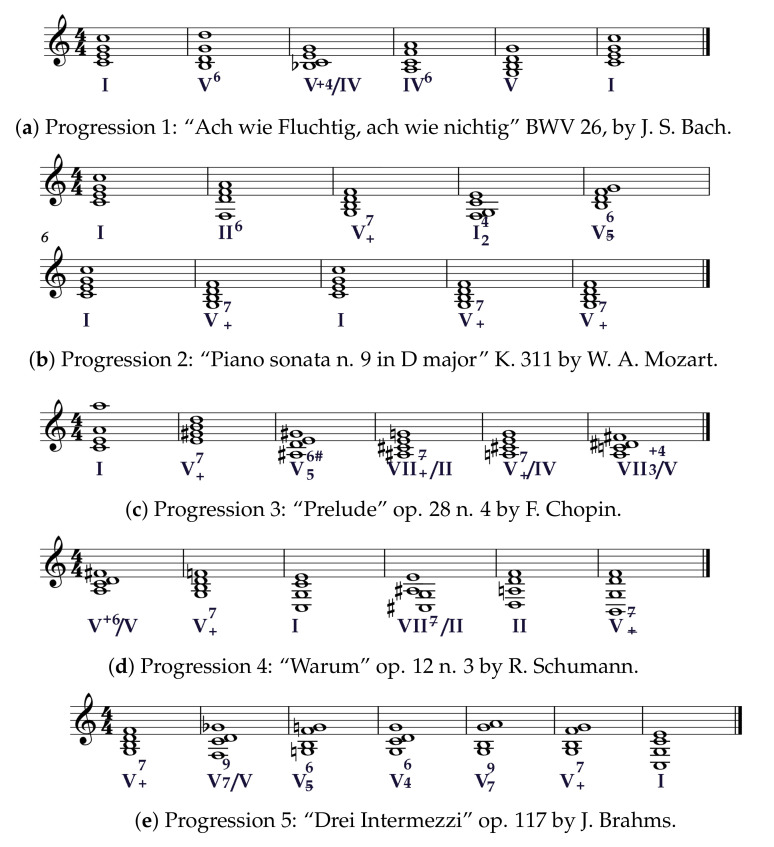

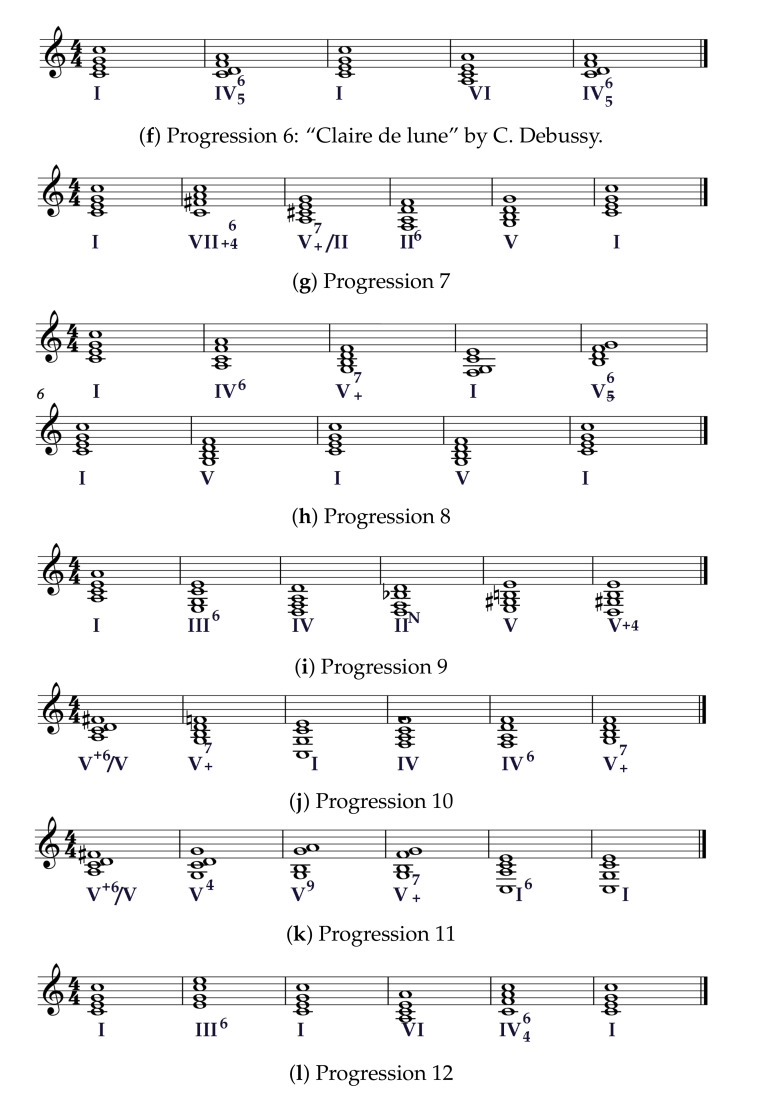
Scores of the chord progressions created for the experiments.
